# Aortic shape with high-acute isthmic angle post frozen elephant trunk reconstruction is associated with worse postoperative outcomes: Multisite, principal component analysis, retrospective study

**DOI:** 10.1016/j.xjse.2024.100025

**Published:** 2024-09-07

**Authors:** Michal Schäfer, Jason P. Glotzbach, Sara J. Pereira, Vikas Sharma, Matthew L. Goodwin, Joseph C. Cleveland, Craig H. Selzman, Adam Carroll, Alex J. Barker, Muhammad Aftab, T. Brett Reece

**Affiliations:** a Division of Cardiothoracic Surgery, University of Utah Health, Salt Lake City, Utah; b Division of Cardiothoracic Surgery, University of Colorado Denver | Anschutz Medical Campus, Aurora, Colo; c Department of Radiology, University of Colorado Denver | Anschutz Medical Campus, Aurora, Colo

**Keywords:** aortic shape, frozen elephant trunk, aortic dissection

## Abstract

**Objective:**

Aortic shape and geometry after surgical reconstruction have been shown to be important determinants of flow hemodynamics, aortic remodeling, and clinical outcomes. In this study, we investigated aortic shapes post-frozen elephant trunk (FET) reconstruction using statistical shape modeling by principal component analysis and correlated discovered shape variations with the postoperative outcomes.

**Methods:**

Patients from 3 institutions who underwent computed tomography (CT) angiography post-FET aortic reconstruction for type A dissection or ascending thoracic aortic aneurysms were included. A 3-dimensional aortic model was generated from CT angiography, and the aortic centerline was calculated for each subject. Principal components describing aortic shape variations were then correlated with the cumulative rate of postoperative aortic events requiring intervention.

**Results:**

A total of 135 subjects across 2 sites with postoperative CT and surveillance data were included in the study. Identified first principal component accounting for 31.6% of geometric shape variation in the entire cohort described angle variability at the region of the aortic isthmus. On the basis of the degree of isthmic angulation quantitatively defined by the principal component score, we separated the cohort into high and low isthmic angle groups. Patients with high isthmic angle had greater 5-year freedom from aortic events with 49.5% compared with 78.6% in the low isthmic angle group (*P* = .024).

**Conclusions:**

Aortic shape variation defined by the acute angle of the aortic isthmus has been associated with aortic events and lower freedom from intervention post-FET operation. These results add to the increasing amount of evidence implicating acute isthmic angle from overt downstream aortic remodeling postcomplex aortic repair. Further biomechanical studies are required to identify the mechanistic link between isthmic geometry and patient-specific aortic remodeling.

**Figure:**
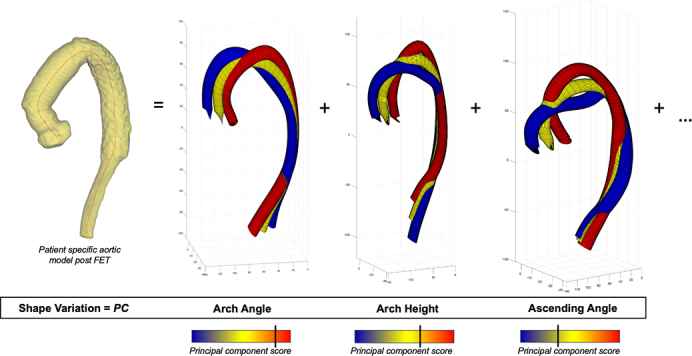
Patient-specific aortic model post-FET derived from different aortic shape variations.

**Central Message**

In this study, we found that aortic variations defined by the acute angle of the aortic isthmus are associated with aortic events and lower freedom from intervention post-FET operation.

**Perspective**

Aortic shape and geometry after surgical reconstruction have been shown to be important determinants of flow hemodynamics, aortic remodeling, and clinical outcomes. Our results add to the increasing amount of evidence implicating acute isthmic angle from overt downstream aortic remodeling postcomplex aortic repair.

Frozen elephant trunk (FET) remains a well-established repair technique for patients with type A acute aortic dissection (TAAD) or complex ascending aortic and arch aneurysms.^1^ The method is ideal for more extensive pathology propagating beyond the subclavian artery and is thought to favor more optimal aortic remodeling.^2^ Whereas advancements have been made in surgical techniques and postoperative care, less progress has been made in understanding and reducing long-term aortic complications. The mid-to long-term reports in studies on the incidence of aortic events postprimary surgical repair vary, ranging from ∼16% to 47% and exceeding 50% after 30 months.^3^, ^4^, ^5^ Current surveillance techniques describe aortic remodeling postdissection repair in terms of true lumen and false lumen (FL) size parameters or aortic growth.^6^, ^7^, ^8^ However, this approach is typically limited to 2-dimensional cross-sectional measurements despite the complex postsurgical 3-dimensional (3D) aortic shape remodeling.

The shift toward more comprehensive imaging analysis considering the effect of aortic shape on hemodynamics has already occurred in studies predicting the rupture and dissection risk in surgically naïve patients with ascending thoracic aortic aneurysms.^9^^,^^10^ Most recent studies have begun to explore the application of machine learning and statistical shape-modeling techniques to investigate in an unbiased fashion aortic geometric parameters and their clinical predictive potential.^11^^,^^12^ Specifically, principal component analysis (PCA) has emerged as a technique capable of describing aortic shape variants in large datasets of 3D aortic models after surgical repair. Importantly, the described aortic shape variants and their contributions to patient-specific anatomies have been associated with hemodynamic and clinical outcomes.^13^^,^^14^ However, large-scale studies investigating the association between postsurgical aortic shape and the long-term FET outcomes are missing.

Consequently, the purpose of this study was to investigate multi-institutional 3D aortic shape data obtained as a part of routine postoperative imaging. Specifically, we hypothesized that aortic shape variants post-FET identified by PCA will be associated with clinical aortic events and outperform standard clinical and pre-operative parameters.

## Methods

This retrospective multisite, compliant study was approved by the institutional review boards at the University of Colorado (COMIRB#17-0198, approval date February 6, 2017; Aurora, Colo; site 1) and the University of Utah (CTSI2163, approval date April 9, 2024, Salt Lake City, Utah; site 2), approved at both sides with approved waiver of informed consent. We included patients who underwent the FET repair for TAAD or had an elective repair of complex aortic arch aneurysm and received postoperative computed tomography (CT) angiography before discharge from the hospital. Patients who died before discharge from the hospital, did not underwent postoperative imaging within 1 month after the surgery, or did not have a clinical follow-up at 1 of the 2 institutions were excluded. All selected patients underwent the FET with specific aortic repairs including either hemiarch repair, zone 2 repair, or total arch replacement.

### Aortic Imaging: Preprocessing

**Figure 1:**
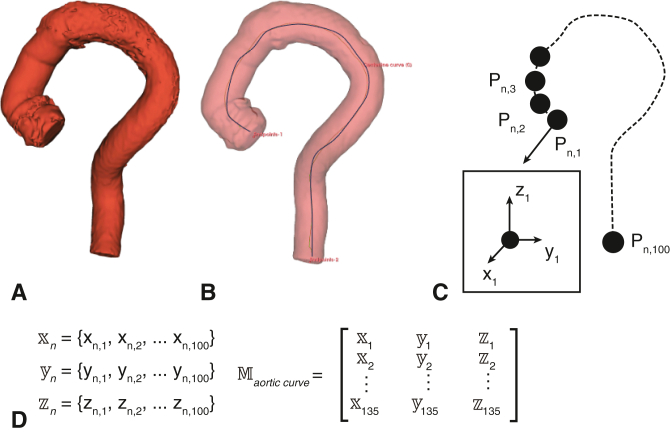
Aortic shape postprocessing scheme. A, Aortic models segmented from the postoperative computed tomography angiography images were preprocessed in standardized fashion by trimming coronary arteries, arch vessels, and were extended distally to the level of the thoracoabdominal junction. B, Thereafter, aortic models were smoothed and aortic centerlines representing the aortic curvature were calculated. C, The centerline was then calibrated to contain 100 specific equally spaced coordinate points along its length. Each coordinate point was defined by its Cartesian coordinates. D, A patient (n = 1:135)-specific set of coordinates was then used to construct the final aortic curve matrix (***M***) analyzed by the principal component analysis.

In both institutions, the postoperative imaging protocol consisted of the thin-slice CT angiography sequence with triple-phase protocol. All images and aortic shape analyses were conducted as described previously.^14^ To summarize, all CT datasets were uploaded to the free open-source software 3D Slicer (version 5.6.1 or newer) to undergo semiautomatic segmentation to yield aortic model. To achieve maximum standardization, we elected to remove all head and neck vessels from the model and considered only the residual true lumen for the shape purposes. The proximal end of the aortic model was defined by the aortic annulus, whereas the distal end was delineated by the thoraco-abdominal junction (Figure 1).

Thereafter, the generated aortic models were preprocessed to develop a standardized aortic centerline that would serve as an input for the PCA. These centerlines consist of a series of coordinates describing the 3D position of iterative points along the aortic length. The standardization preprocessing specifically consisted of (1) patient-specific aortic length adjustment to the entire population mean aortic length, (2) centerline point interpolation to yield 100 coordinate points along each patient specific aortic centerline, and (3) rigid registration to optimally align individual aortic centerlines for an objective shape comparison in a fixed space. A specific detail behind each preprocessing step have been described previously.^14^ The final former analysis of all aortic centerlines consisted of analyzing a large matrix containing patient specific coordinates along the aortic length.

### PCA of the Aortic Shape

**Figure 2:**
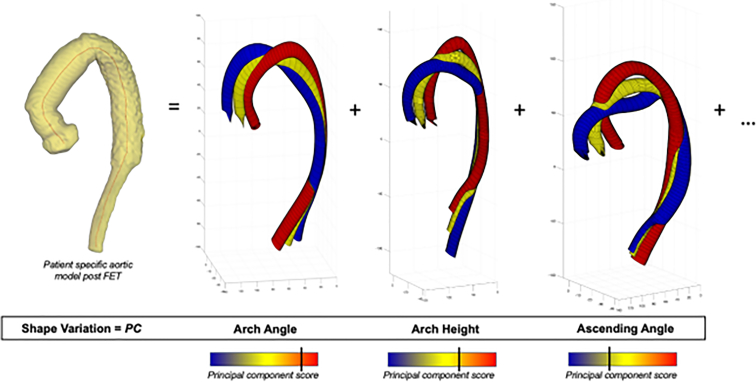
A hypothetical composition of the patient-specific aortic model post-frozen elephant trunk. Every single model can be eventually constructed from discovered principal components (*PC*) representing aortic shape variations and the degree of these shape variation (principal component score).

PCA is a dimensionality reduction technique frequently applied in a statistical shape analysis or machine learning algorithms. As such, the technique in unbiased fashion explains the largest variation within the dataset. In the context of the aortic shape analysis, the PCA will describe the most frequent shape patterns (eg, arch angulation, ascending aortic curvature) and will assign them overall importance in the entire set of aortic shapes. Furthermore, every patient-specific aortic model can then be described in terms of shape variation (principal component) and the degree of that variation (principal component score). The hypothetical scenario of a composition of a patient-specific model on the basis of observed aortic shape variation and their specific scores is graphically depicted in Figure 2.

### Statistical Analysis

Statistical analyses were performed in Prism (version 10.1.1; GraphPad Software). All considered variables were checked for the distributional assumption of normality using normal plots, in addition to Kolmogorov-Smirnov and Shapiro-Wilks tests. All normally distributed variables were reported as means ± standard deviation or as median with corresponding interquartile ranges. Demographic and clinical characteristics between groups (events vs no events) were compared using the Student *t* test for normally distributed continuous variables. The Mann-Whitney *U* test was used for non-normally distributed variables, and χ^2^ test was used for categorical variables.

Pre- and perioperative variables and aortic shape-based PC scores were subjected to standard univariable logistic regression. Receiver operating characteristic curves were generated, and c-statistic values were calculated for each variable. We further investigated the effect of standard pre- and perioperative variables and PCA-derived shape scores on predicting aortic events. In model 1, we considered all variables considered in univariable analysis to multivariable analysis. Reduction-optimized model 2 was developed only to include variables predictive of aortic events in multivariable analysis. Variables included in multivariable models were checked for collinearity using variance inflation factors (values in the range of 1-3 were considered) and a correlation matrix.

For the principal component variables that were found to be significantly associated with aortic events, Kaplan-Meier survival curves were constructed with a specific log-rank test with the population divided by receiver-operating characteristics to find the most optimal cut-off values. The proportionality assumption was tested using Schoenfeld residuals and graphically using log-log plots. We have added this description to our analysis. All patients were followed up to the particular aortic event (terminal event or event requiring further intervention) or the end of the study (December 31, 2023). Significance was determined by an alpha value of 0.05.

## Results

**Figure 3:**
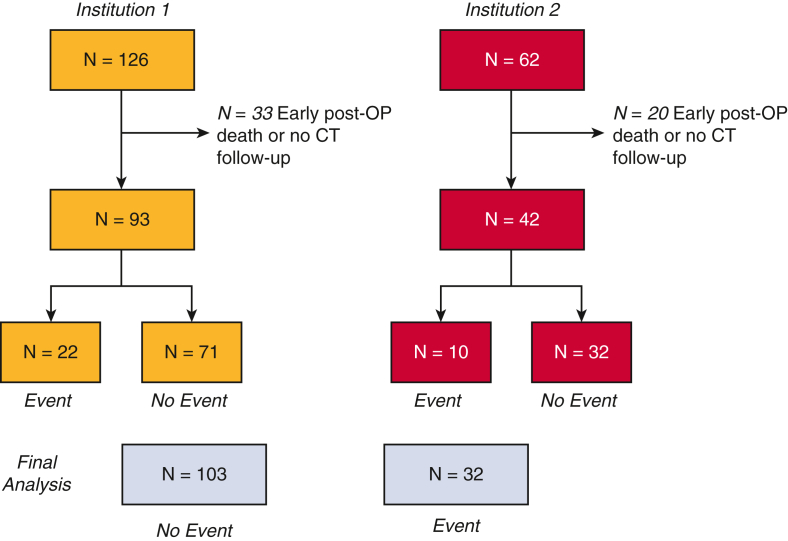
Overall study diagram depicting the institution specific numbers, excluded patients, and final categorization and sample sizes. *post-OP*, Postoperative; *CT*, computed tomography.

In total, 188 patients from both institutions who underwent FET were identified and evaluated for study inclusion. Fifty-three patients were excluded for the early postoperative death or loss to follow-up (Figure 3). Consequently, 135 were considered in study (institution 1, n = 93; institution 2, n = 42), and 32 patients (23.7%) experienced an aortic event (institution 1: n = 22 [23.6%]; institution 2: n = 10 [23.8%]). Aortic events included death from aortic rupture or end-organ ischemia (n = 4), gradual thoracoabdominal aortic degeneration with persistent FL flow (n = 12), new growing arch or thoracoabdominal aneurysm (n = 5), new type B dissection (n = 4), new aortic root dissection or pseudoaneurysm (n = 3), and type 3 endoleak requiring further thoracic endovascular aortic repair (n = 4).

Table 1 summarizes the patient characteristics and perioperative variables. The indication for the aortic repair with FET was primarily TAAD, accounting for 71.8% in patients with no event and 75.0% of patients with an event (*P* = .588). There were no differences between the 2 groups regarding proportion of patients who underwent previous aortic surgery (no event 24 [25.8%] vs event 8 [30.8%], *P* = .843). Furthermore, there were no differences in perioperative need for aortic valve repair/replacement (no event 30 [29.1%] vs event 11 [34.4%], *P* = .318) or in aortic root intervention (no event 33 [32.0%] vs event 9 [28.1%], *P* = .676). The only difference between the 2 groups was observed in greater prevalence of connective tissue disease among the patients with an aortic event (no event 4 [3.8%] vs event 5 [16.1%], *P* = .020).

**Table 1:** Patient characteristics and perioperative variables

	No event (n = 103)	Events (n = 32)	*P* value
Age, y	58.5 ± 12.1	57.2 ± 15.1	.654
Sex (female %)	30 (29.1%)	5 (15.6%)	.128
Smoking	23 (22.3%)	9 (28.1%)	.501
Hypertension	41 (39.8%)	16 (50%)	.308
Diabetes	19 (18.4%)	8 (25.0%)	.518
Pathology			
Type A aortic dissection	74 (71.8%)	24 (75.0%)	.588
Ascending aortic aneurysm	32 (31.1%)	8 (25.0%)	.511
Perioperative variables			
Previous aortic surgery	24 (25.8%)	8 (30.8%)	.843
Valve intervention	30 (29.1%)	11 (34.4%)	.318
Root intervention	33 (32.0%)	9 (28.1%)	.676
Connective tissue disease	4 (3.8%)	5 (16.1%)	.020
Principal component analysis			
PC1 score	3.4 ± 49.0	−18.9 ± 43.7	.018
PC2 score	−1.5 ± 45.4	4.9 ± 53.8	.542
PC3 score	1.5 ± 27.2	−4.7 ± 31.6	.326

Data are reported as mean ± standard deviation. Reported *P* value represent Student *t* test or χ^2^ test. *PC*, Principal component.

**Figure 4:**
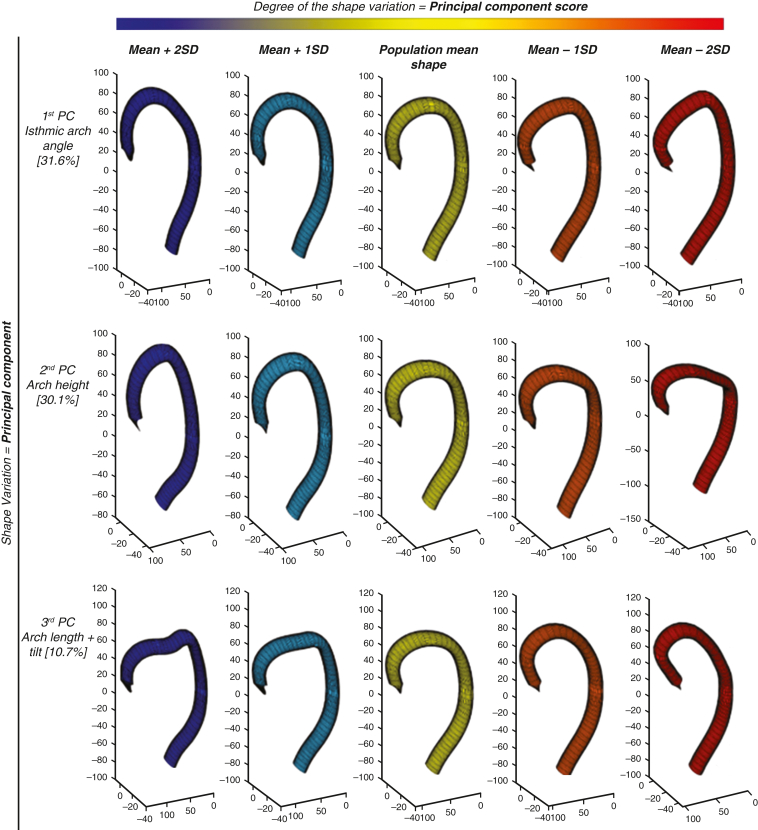
Graphical depiction of the principal component analysis result describing here only the first 3 most important principal components (*PC*). These PCs are describing the variation in (1) isthmic arch angulation (first PC), responsible for 31.6% of shape variation among all considered patients, (2) arch height (second PC), accounting for 30.6% of all shape variation, and (3) arch length combined with tilt (third PC), responsible for 10.7% of all aortic shape variations.

The summary of the PCA of aortic shapes is summarized in Figure 4. The first 3 primary shape variations (principal components) accounted cumulatively for 71.3% of the total variation described in the entire dataset. These shape variations described alterations in (1) aortic angle at the isthmic region (31.6% of the total variation), (2) arch height (30.1%), and (3) arch length combined with tilt (10.7%). Comparison of the principal component scores describing variation along each shape variation is depicted in Table 1. The first principal component score was decreased (more negative) in patients who experienced aortic events (no event, 3.4 ± 49.0, vs event, −18.9 ± 43.7, *P* = .018), signifying greater propensity to have more acute isthmic angle in these patients. There were no differences in the second (no event, −1.5 ± 45.4, vs event 4.9 ± 53.8, *P* = .542) and the third (no event, 1.5 ± 27.2, vs event, −4.7 ± 31.6, *P* = .326) principal component scores.

**Figure E1:**
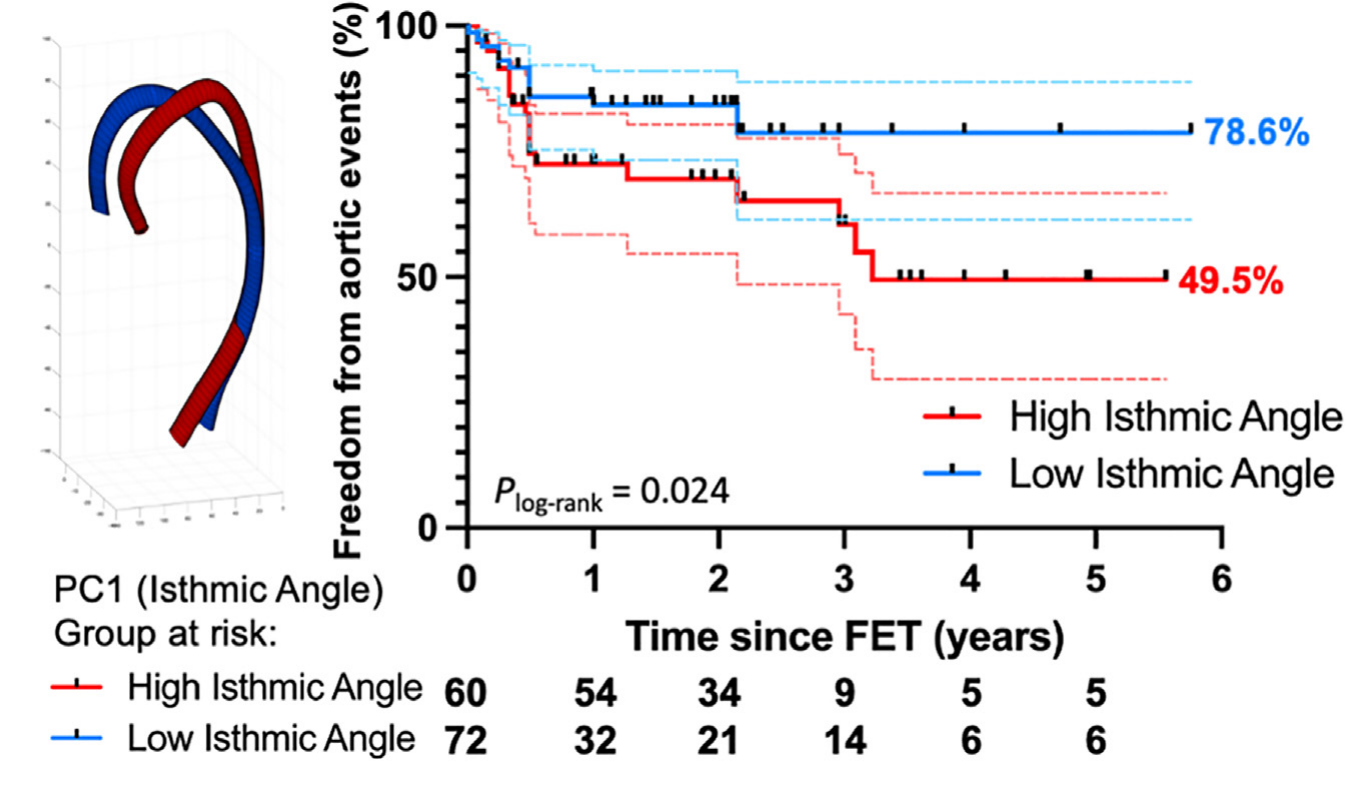
Kaplan-Meyer plot depicts freedom from all aortic events. Patients with high or acute isthmic angle (principal component score <0) had lower freedom from aortic events (*red line*) when compared with patients with low or smoother isthmic angle (principal component score >0) (*blue line*). *PC*, Principal component; *FET*, frozen elephant trunk.

The median time to follow-up was 12.9 months (range, 0.2-69 months). The Kaplan-Meier curves for patients with acute isthmic angle (principal component 1 score values <0) and low acute isthmic angle (principal component 1 score values >0) are shown in Figure E1. Patients with high isthmic angle had greater 5-year freedom from aortic events with 49.5% compared with 78.6% in the low isthmic angle group (*P* = .024).

**Figure E2:**
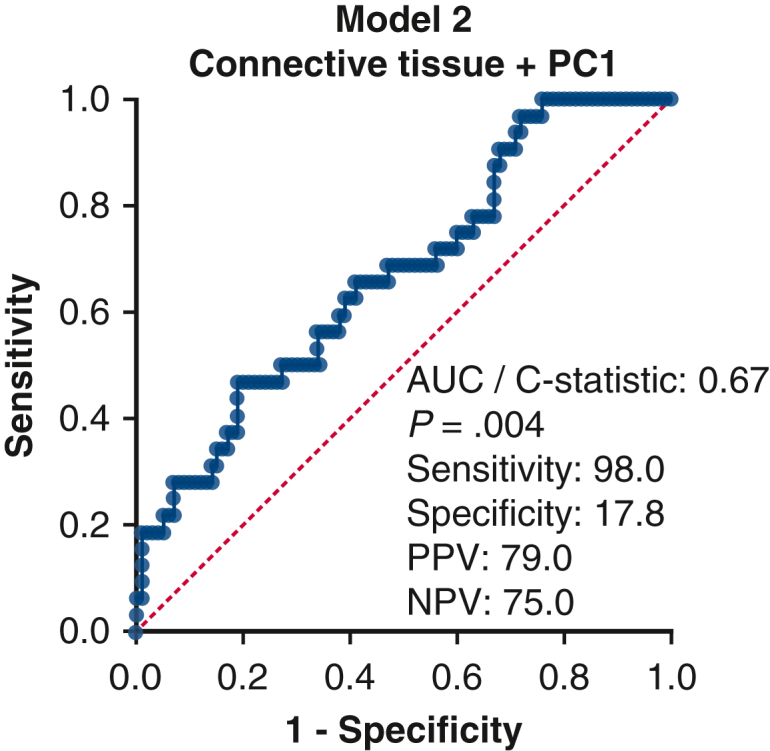
The receiver operating characteristic curve for optimized Model 2 involves 2 variables: a history of connective tissue disease and the principal component (*PC*) 1 score. *AUC*, Area under the curve; *PPV*, positive predictive value; *NPV*, negative predictive value.

When exposed to univariable logistic regression analysis (Table 2), 2 variables were predictive of aortic events, including the history of connective tissue disease (*P* = .041) and the principal component 1 score (*P* = .005). Multivariable analysis is summarized in Table 3. When considering all variables, 2 variables were significantly associated with the aortic events, the history of connective tissue disease (*P* = .046) and the principal component 1 score (*P* = .007) scores were associated with the periprocedural complications, with the overall model yielding a C-statistic of 0.71 with a sensitivity of 95.0% and specificity of 18.7%. An optimized reduced model considering only the history of the connective tissue disease and the principal component 1 score revealed a C-statistic of 0.67 with a sensitivity of 98.0% and specificity of 18.7%, with a positive predictive value of 79.0% and negative predictive value of 75.0% (Figure E2).

**Table 2:** Univariable logistic regression to predict aortic events

	OR (95% CI)	AUC/c-index	*P* value
Age	0.99 (0.96-1.02)	0.51	.611
Sex	0.45 (0.14-1.17)	0.57	.129
Type A aortic dissection	1.16 (0.48-3.04)	0.51	.740
Previous aortic surgery	1.56 (0.69-3.56)	0.55	.271
Valve intervention	1.28 (0.53-2.96)	0.53	.565
Root intervention	0.83 (0.33-1.95)	0.52	.680
Connective tissue disease	3.07 (1.02-10.0)	0.58	.041
PC1 score	0.98 (0.97-0.99)	0.64	.005
PC2 score	1.00 (0.99-1.01)	0.55	.504
PC3 score	0.99 (0.98-1.02)	0.51	.284

Data represented as odds ratios (OR) with corresponding 95% confidence interval (CI). *AUC*, Area under the curve; *PC*, principal component.

**Table 3:** Multivariable logistic regression model to predict aortic events

	Model 1		Model 2	
	OR (95% CI)	*P* value	OR (95% CI)	*P* value
Age	1.01 (0.97-1.05)	.658		
Pathology—type A dissection	0.72 (0.25-1.90)	.518		
Previous aortic surgery	1.34 (0.54-3.30)	.521		
Valve intervention	0.81 (0.32-2.09)	.657		
Connective tissue disease	3.13 (1.06-14.6)	.046	2.99 (0.87-10.1)	.075
PC 1	0.98 (0.97-0.99)	.007	0.98 (0.97-0.99)	.006
PC 2	1.00 (0.99-1.01)	.814		
PC 3	0.99 (0.98-1.01)	.487		

Model parameters		
C-statistic	.71	.67
Sensitivity, %	95	98
Specificity, %	18.7	18.7
Positive predictive value, %	78.5	79
Negative predictive value, %	54.5	75
ROC *P* value	<.001	.004
Hosmer-Lemeshow *P* value	.915	.438

Data represented as model specific odds ratios (OR) with corresponding 95% confidence interval (CI). *ROC*, Receiver operating characteristic.

## Discussion

The FET technique used for the treatment of TAAD or complex arch aneurysms has been shown to have favorable remodeling effects on distal aortic segments, yet up to 40% of patients might require additional interventions.^6^^,^^15^ Multiple studies have attempted to predict the rate of aortic events requiring intervention using preoperative aortic dimensions, perioperative variables, or postoperative remodeling of the FL. This task is inherently challenging, given the heterogeneous pathologic spectrum of the thoracic aortic disease and highly variable clinical presentation and disease extent. Furthermore, postsurgical changes entirely change the aortic phenotype by altering the aortic shape, tissue mechanics, and even blood flow hemodynamic conditions.^16^^,^^17^ In this study, we have attempted to use a novel approach to evaluate postsurgical aortic state by first identifying primary modes of aortic shape using a nonbiased statistical shape tool and second by associating the varying degrees of aortic shapes with subsequent aortic events. Our results suggest that acute angulation at the region of the aortic isthmus post-FET is associated with lower freedom from subsequent aortic events. Further understanding of the resultant 3D aortic shape may help to complement standard surveillance cross-sectional aortic dimensional parameters and possibly guide the initial surgical approach.

The relationship between the shape of the aortic curvature and hemodynamic conditions has already been described in patients postcoarctation repair or Norwood reconstruction.^13^^,^^18^ Specifically, abrupt diameter changes along the aortic curvature have been associated with abnormal wave reflections and hemodynamically less efficient flow patterns. Considering the adult patient population and the thoracic aortic disease, recent comprehensive shape analyses have described aortic variants associated with the greater risk of rupture and dissection in surgically naïve patients with ascending thoracic aortic aneurysms.^9^^,^^10^ This finding was indirectly supported by demonstrating that the length of the 3D centerline of the ascending aorta can serve as a potential risk criterion for pre-emptive operative repair and outperforms standard aortic diameter measurements.^7^ Similarly, shape-based features were recently described to predict the growth of the ascending aortic aneurysms.^19^ Statistical shape analysis methods also differentiated aneurysmal shape patterns between the patients with tricuspid and bicuspid aortic valves.^20^ In general, aortic shape variants have been invariably linked to different pathologies, aneurysmal disease progression, and also to postoperative hemodynamic outcomes.

In this study, we are specifically describing the association between acute isthmic angle and a greater rate of aortic events. The biomechanical link is currently unknown, but we speculate that the acute angulation between the aortic arch and the descending aorta strengthened by the stent-graft results in local impedance mismatch introducing abnormal hemodynamic wave reflections as well as abnormal distal wave propagation beyond the isthmus. The site of impedance mismatch also generates backward compression waves traveling toward the heart and elevating left ventricular afterload and mechanical stress on the aortic root, possibly explaining proximal aortic complications. Such pathophysiology can be observed in patients with coarctation, postarterial switch operation, or gothic arch variants.^21^, ^22^, ^23^ Gothic-type aortic arch has also been described as an independent risk factor for developing long-term hemodynamic sequelae such as systemic hypertension.^24^^,^^25^ Lastly, acute or sharp angulation at the distal aortic arch is associated with an increased incidence of acute type B aortic dissections.^26^ In our study, PCA discovered in an unbiased fashion aortic isthmic angulation as a critical shape variant post-FET. Principal component scores describing the degree of this shape variation were then predictive of aortic events requiring further intervention. Notably, the principal component score describing the degree of isthmic angulation was, along with the history of connective tissue disease, an independent predictor of aortic events in multivariable analysis. The nature of the presenting pathology and the extent of the surgical repair were not associated with the subsequent aortic events, possibly suggesting the critical role of resultant surgical repair. However, we warrant a cautious interpretation of these results, as our shape analysis did not consider the nature and extent of the FL and final position of head and neck vessels. Our and previously discussed results offer compelling evidence regarding the mechanism by which an angulated distal portion of the arch likely mediates downstream hemodynamic forces. Modification of the proximal stent graft using fenestration or surgical attempt to create a smooth curvature at the site of the distal arch anastomosis gradually ensuring might alleviate mechanical tensile and flow-mediated wall shear stress at the isthmic region. However, additional biomechanical analyses based on patient-specific pathologies need to be performed to understand the relationship between postsurgical geometry and aortic remodeling fully.

### Limitations

We would like to acknowledge several limitations pertinent to this study. This was a multi-institutional retrospective study that involved only patients undergoing FET with different types of the aortic surgery including hemiarch replacement, zone 2 arch replacement, or total arch repair. Consequently, proximal flow hemodynamic conditions differed in these patients and should be considered in future investigations. Furthermore, our geometric analysis did not include arch vessels, which can further effect arch hemodynamics and downstream flow patterns.^27^ The only additional aortic characteristic associated with aortic events was the presence of connective tissue disorder, which is inherently associated with a greater risk of postoperative complications.^2^ An additional limitation of the principal component-derived scores is that they currently have only limited clinically applicable use unless 3D shape analysis is performed on every patient. Our next investigation involves converting the principal component score to the clinically translational parameter or standardized 2-dimensional measurement. We are currently evaluating what standardized set of views would have to be applied to achieve reproducible isthmic angle measurements. Finally, we had to exclude the residual FL in relevant patients to investigate the aortic curvature in a standardized fashion.

## Conclusions

Aortic shape variation defined by the acute angle of the aortic isthmus has been associated with aortic events and lower freedom from intervention post-FET operation. These results add to the increasing amount of evidence implicating acute isthmic angle from overt downstream aortic remodeling post complex aortic repair. The PCA used in this study identified this shape variation across patients treated in 2 institutions and highlights the importance of postsurgical aortic surveillance using comprehensive 3-dimensional analysis. Further biomechanical studies are required to identify the mechanistic link between isthmic geometry and patient-specific aortic remodeling.

## Conflict of Interest Statement

The authors reported no conflicts of interest.

The *Journal* policy requires editors and reviewers to disclose conflicts of interest and to decline handling or reviewing manuscripts for which they may have a conflict of interest. The editors and reviewers of this article have no conflicts of interest.
